# Constitutive and evoked release of ATP in adult mouse olfactory epithelium

**DOI:** 10.1515/biol-2022-0811

**Published:** 2024-01-16

**Authors:** Sébastien Hayoz, Cuihong Jia, Colleen Cosgrove Hegg

**Affiliations:** Department of Physiology, University of Arizona, Tucson, Arizona 85724, USA; Department of Biomedical Sciences, East Tennessee State University, Johnson City, Tennessee 37614, USA; Department of Pharmacology and Toxicology, Michigan State University, East Lansing, Michigan 48824, USA

**Keywords:** primary cell cultures, OP6 cells, confocal imaging, immunohistochemistry, luciferin–luciferase assay

## Abstract

In adult olfactory epithelium (OE), ATP plays a role in constant cell turnover and post-injury neuroregeneration. We previously demonstrated that constitutive and ATP-evoked ATP release are present in neonatal mouse OE and underlie continuous cell turn-over and post-injury neuroregeneration, and that activation of purinergic P2X_7_ receptors is involved in the evoked release. We hypothesized that both releases are present in adult mouse OE. To study the putative contribution of olfactory sensory neurons to ATP release, we used olfactory sensory neuronal-like OP6 cells derived from the embryonic olfactory placode cells. Calcium imaging showed that OP6 cells and primary adult OE cell cultures express functional purinergic receptors. We monitored ATP release from OP6 cells and whole adult OE turbinates using HEK cells as biosensors and luciferin–luciferase assays. Constitutive ATP release occurs in OP6 cells and whole adult mouse OE turbinates, and P2X_7_ receptors mediated evoked ATP release occurs only in turbinates. The mechanisms of ATP release described in the present study might underlie the constant cell turn-over and post-injury neuroregeneration present in adult OE and thus, further studies of these mechanisms are warranted as it will improve our knowledge of OE tissue homeostasis and post-injury regeneration.

## 1 Introduction

The olfactory epithelium (OE) comprises several distinct cell types [1]. The OE is pseudostratified: the cell bodies of the glia-like sustentacular cells are located in the apical layer, the cell bodies of the olfactory sensory neurons (OSN) that detect odorants are located in the middle layer, and progenitor/stem cells that allow neuroregeneration and constant cell turn-over are located in the basal layer. The OE also houses in the apical layer a population of microvillous cells whose exact function is still unclear. Cells of all OE layers express both P2X purinergic receptors and P2Y purinergic receptors [2–4]. P2X are ionotropic receptors and P2Y are metabotropic receptors; their activation by ATP plays an essential role in mechanisms involving ATP as a neurotransmitter [5]. Several subtypes of P2X and P2Y purinergic receptors are present in mouse OE [2–4]. Purinergic signaling has been well studied in the vertebrate olfactory system [6] and ATP has been shown to modulate responses to odorants [7,8]. Moreover, in vertebrate OE, purinergic signaling has been shown to be involved in communication between the different cell types [2,9,10]. Purinergic signaling also plays a very important injury-related role in mouse OE. Indeed, ATP has both protective and proliferative effects following injury [11,12]. One ATP mechanism of action is direct stimulation of the purinergic receptors located on the OE basal progenitor cells [2,13,14]. ATP also indirectly induces the upregulation and release of other neurotrophic factors such as neuropeptide Y and fibroblast growth factor 2 [15–18], leading to cell proliferation in adult mouse OE via activation of neuropeptide Y and fibroblast growth factor receptors [16–18]. Therefore, study of the mechanisms underlying ATP release is essential to fully understand how the OE maintains tissue homeostasis and recovers from injury.

ATP release has been well documented in several organ systems and sensory systems including sensory axons in the olfactory bulb [5,19]. ATP-induced ATP release has been demonstrated in endothelial cell culture [20,21], in taste bud cells [22] and in cultured astrocytes [23]. Moreover, we showed that both ATP-evoked ATP release and constitutive ATP release occur in neonatal mouse OE through numerous mechanisms [4]. The constitutive release of ATP likely occurs in physiological, i.e., injury-free conditions, to promote constant cell turnover and tissue homeostasis in the OE. The evoked ATP release, activated by purinergic receptor stimulation, is likely triggered by ATP leaking from damaged cells, inducing subsequent post-injury OE regeneration. When we previously investigated the mechanisms underlying the evoked ATP release in mouse neonatal OE, we became interested in one sub-type of P2X purinergic receptors, P2X_7_. This sub-type, encoded by the P2rx7 gene, has unique properties. Activated P2X_7_ receptors form large pores leading to either influx or efflux of ions and other molecules [24–26]. Moreover, it has been shown in several cell types that activated P2X_7_ receptors can also form complexes with pannexin1 channels, which can lead to efflux of ATP or other molecules [27–32]. We indeed showed that one of the mechanisms involved in ATP-evoked release of ATP in neonatal mouse OE is activation of P2X_7_ receptors and subsequent formation of P2X_7_ large pores and/or P2X_7_–pannexin complexes [4]. We also showed that stimulation of P2Y purinergic receptors also contribute to the evoked release of ATP. We hypothesized that similar mechanisms of constitutive and evoked ATP release are present in adult OE. To specifically investigate the putative role of OSN in ATP release, we used the OP6 cell line, a clonal temperature-sensitive cell line derived from the E10 mouse olfactory placode. Undifferentiated OP6 cells are similar to immature OSN and differentiated OP6 cells are similar to mature OSN, as described by Illing et al. [33]. We first used calcium imaging to show the presence of functional purinergic receptors in OP6 cells and adult OE cells, a requirement to study purinergic receptor-mediated ATP release in adult OE. Then, using luciferin–luciferase assays and human embryonic kidney (HEK-293) cells as ATP biosensors, we showed that constitutive and evoked ATP release both occur in whole adult OE turbinates, but that both immature and mature OP6 cells only have a constitutive release of ATP. We also showed that P2X_7_ purinergic receptors are involved in the evoked release of ATP. We conclude that (a) functional purinergic receptors are present in adult mouse OE and activation of the P2X_7_ receptor subtype is sufficient for evoked release of ATP, (b) constitutive ATP release is also present in adult mouse OE, and (c) OSN only constitutively release ATP, the evoked release of ATP thus originating most likely from other cell types. However, it does not exclude that the other OE cell types might constitutively liberate ATP as well, and that activation of other P2X and/or P2Y receptor subtypes might contribute to the evoked release of ATP.

## 2 Materials and methods

### 2.1 Chemicals

Fluo-4 AM was purchased from Invitrogen (Carlsbad, CA, USA). ATP determination kits were purchased from Molecular Probes (Eugene, OR, USA). All other chemicals were purchased from Sigma-Aldrich (St Louis, MO, USA).

### 2.2 Solutions

Ringer’s solution contained (in mM) 140 NaCl, 5 KCl, 1 MgCl_2_ 6H_2_O, 2 CaCl_2_, 10 HEPES, and 10 glucose (pH 7.4). Calcium-free Ringer’s solution contained (in mM) 140 NaCl, 5 KCl, 10 HEPES, 4 EGTA, and 20 glucose (pH 7.4). Concentrated stock solutions of ATP, 2′(3′)-*O*-(4-benzoylbenzoyl)adenosine 5′-triphosphate (Bz-ATP), and *Clostridium difficile* toxin A were made in Ringer’s solution, stored at −20°C and, on the day of the experiment, diluted to concentration values indicated in the text. Fresh stock solutions of carbenoxolone and probenecid dissolved in Ringer’s solution were made on the day of the experiment and diluted to concentration values indicated in the text.

### 2.3 Olfactory turbinates dissection

All animal procedures were approved by Michigan State University’s IACUC, and all applicable guidelines from NIH were followed. Swiss Webster mice around 3–8 weeks old (Charles River, Portage, MI) were anesthetized (i.p. ketamine/xylazine 65/5 mg/kg body weight, respectively) and sacrificed by decapitation. We used Swiss Webster mice to be consistent with our previous study that characterized constitutive and evoked ATP releases in neonatal OE, and that also used Swiss Webster mice [4]. Fur and skin were removed to expose the bone and the skull cut in half with a razor blade to expose the septum and turbinates. Fine forceps were used to remove the septum and extract the full set of turbinates, identified via the paper and online atlas by Barrios et al. [34]. Turbinates were immediately placed in Ringer’s solution (see Section 2.2) and used either for preparing cell cultures (see Section 2.4) or for luciferin–luciferase assays (see Section 2.6).


**Ethical approval:** The research related to animal use has been complied with all the relevant national regulations and institutional policies for the care and use of animals, and has been approved by the Michigan State University’s IACUC.

### 2.4 Cell cultures

Primary cell cultures were prepared from olfactory turbinates collected from 3 weeks old Swiss Webster mice (see Section 2.3) and dissociated as described previously [35]. Primary cultures were grown on coverslips coated with concavalin A (10 mg/mL; Sigma type IV) and used at 7–8 days *in vitro* (DIV) for calcium imaging or immunohistochemistry. The OP6 cell line, a clonal temperature-sensitive cell line derived from the E10 mouse olfactory placode [33], was generously provided by Dr Mary Lucero from University of Utah. At a permissive temperature (33°C), OP6 cells are undifferentiated and behave like immature OSN. At a non-permissive temperature (39°C), all-trans retinoic acid induces the differentiation into a population of mature bipolar OSN-like cells. OP6 cells were plated onto coverslips coated with laminin (20 μg/mL), grown and/or differentiated as described previously [33], and used at 4–8 DIV for calcium imaging or luciferin–luciferase assays. P2X_2_-transfected HEK-293 cells used as biosensors were generously provided by Dr James Galligan from Michigan State University and were maintained as described in our previous study [4]. Briefly, they were grown in Dulbecco’s modified Eagle’s medium F-12 containing 10% fetal bovine serum, 10% GluMax (Invitrogen, Carlsbad, CA, USA), and penicillin and streptomycin (Invitrogen, Carlsbad, CA, USA; 100 units/mL each). HEK-293 cells were passaged once every 3 days when they reached 90% confluence. Afterward, HEK-293 cells were plated on 35 mm coverslips and maintained at 37°C (5% CO_2_) for 24 h before use in calcium imaging experiments.

### 2.5 Confocal calcium imaging

**Figure 1:**
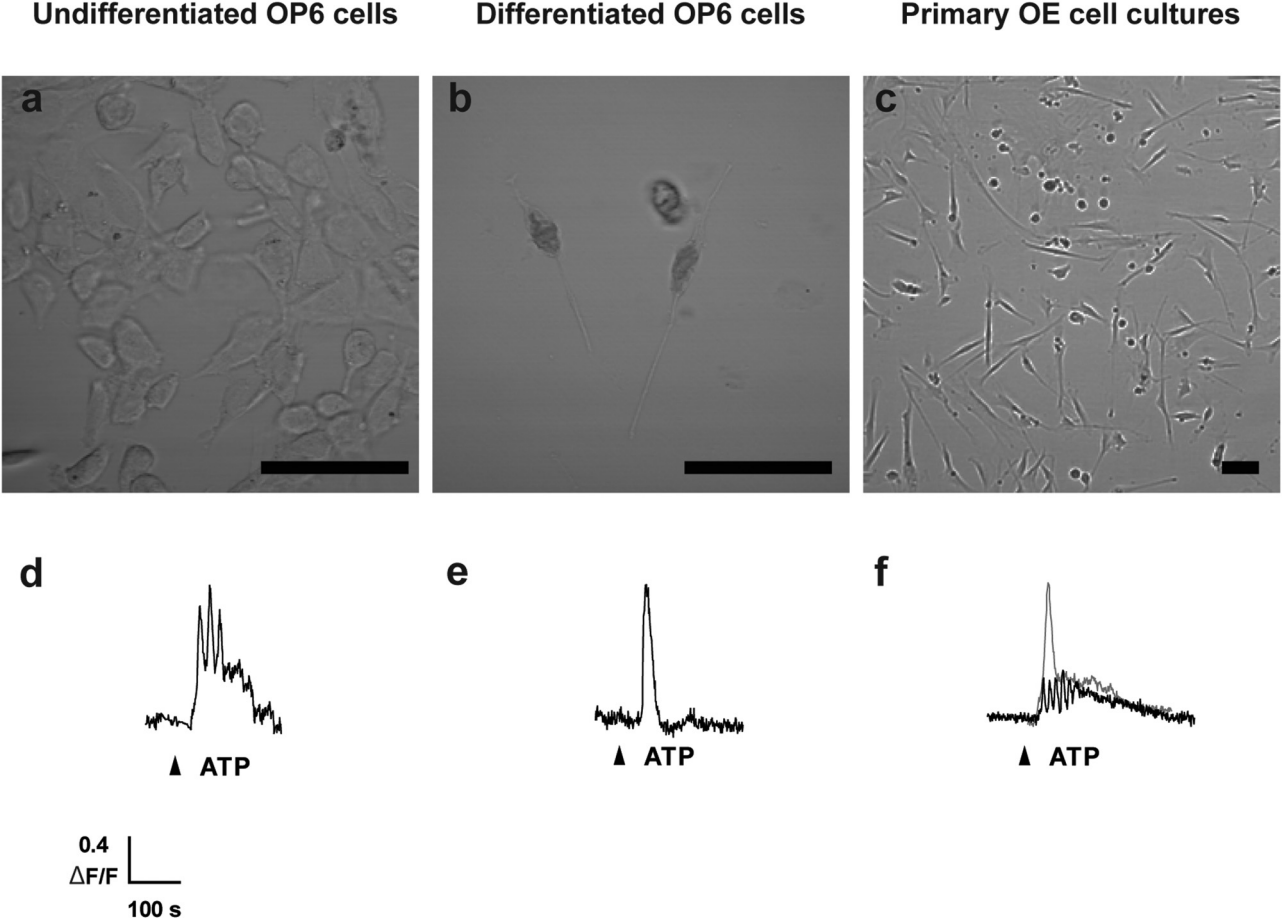
OP6 cells and adult mouse OE primary cell cultures have functional purinergic receptors. (a–c). Representative grayscale images of (a) undifferentiated OP6 cells, (b) differentiated OP6 cells, and (c) 7–8 DIV primary adult OE cell cultures. Scale bar = 50 µm. (d–f) Representative calcium responses; each black or gray trace shows a response recorded in a single cell. Thus, traces from two different cells were superimposed in panel (f). Trace in (d): undifferentiated OP6 cells, trace in (e): differentiated OP6 cells; traces in (f): adult OE primary cell cultures. ▲, application of 50 µM ATP. *n* = 3 coverslips for each experiment.

Live cell confocal imaging of primary OE cell cultures, OP6 cells, and HEK-293 cells, was performed using an Olympus Fluoview 1,000 LSM system. Cells were loaded with fluo-4 AM (Invitrogen, Carlsbad, CA, USA) via incubation for 30 min in Ringer’s solution containing 18 µM of the fluo-4 AM dye. Time series experiments were performed by collecting 256 × 128 pixel images at ≥1 Hz. ATP and Bz-ATP were applied using bath exchange and a 200 µL-volume loop injector (designated ▲ in Figure 1). The fluorometric signals obtained are expressed as relative fluorescence (*F*) change, Δ*F*/*F* = (*F* − *F*_0_)/*F*_0_, where *F*_0_ is the basal fluorescence level (mean *F* of first ten frames). To be considered a true ATP-elicited response, a fluorescence increase had to have a peak normalized value above a cutoff of three times the standard deviation of the baseline. The cutoff was calculated for each individual cell from the baseline points preceding ATP application (imaging of primary OE cell cultures, OP6 cells, or HEK-293 cells in the absence of an OE set of turbinates) or Bz-ATP application (imaging of HEK-293 cells in the presence of a set of turbinates). During each imaging session, at least 30% of the imaged cells responded with calcium increases high enough to be considered true responses. To prevent the fluorescence increase of one cell from being detected by a neighboring cell during analysis (false positive), and only have cells with clearly defined borders when drawing region of interest for data analysis, we purposely imaged regions of the coverslips where we had a low density of cells.

### 2.6 Bioluminescence detection of ATP

ATP was quantified using 0.5 mM d-luciferin and 4 μg/mL luciferase in Ringer’s solution obtained from an ATP determination kit as previously described [4]. We continuously recorded luminescence (in relative light units) with 1-s photon collection intervals, using a Turner TD20/20^n^ luminometer. On the day of the experiment, we made standard curves of ATP (Mg salt) ranging between 10 nM and 5 μM from a 0.5 M ATP stock and recorded them in 200 μL of luciferin and luciferase solution. The ATP standard curves were linear in the range of 10 nM–5 μM (*r* ^2^: 0.93–0.99). We used either whole olfactory turbinates or coverslips plated with OP6 cells. Whole olfactory turbinates were collected from 6 to 8 weeks old Swiss Webster mice as described (see Section 2.3). OP6 cells were either undifferentiated or differentiated. For each individual luciferin–luciferase assay, one set of OE turbinates (either dissected from the left side or the side right of the nose) or one OP6 cell coverslip was placed in a 200 μL bolus of luciferin and luciferase solution in a 35 mm petri dish and ATP release rates were measured for 10 min in presence or absence of inhibitors. Bz-ATP was used to trigger purine-evoked ATP release during the luciferin–luciferase assays since it was the purinergic receptor agonist that elicited the lowest amount of luminescence when applied alone in a bolus of luciferin and luciferase solution, as previously described [4]. Pilot studies showed that the luminescence baseline was stabilized at *t* = 10 min after a sample was put into the luciferin and luciferase solution. Therefore, all data were analyzed at *t* = 10 min. Raw relative light unit values were converted to ATP concentration using the standard curve (relative light units vs ATP concentration [μM]) and interpolation from a linear regression by Prism Software version 9.1.0 (Graphpad Software, San Diego, CA). At the end of each experiment, as a positive control, a bolus of Triton X-100 was added (0.5% final concentration) to release ATP via lysis of the cells.

### 2.7 Immunocytochemistry

**Figure 2:**
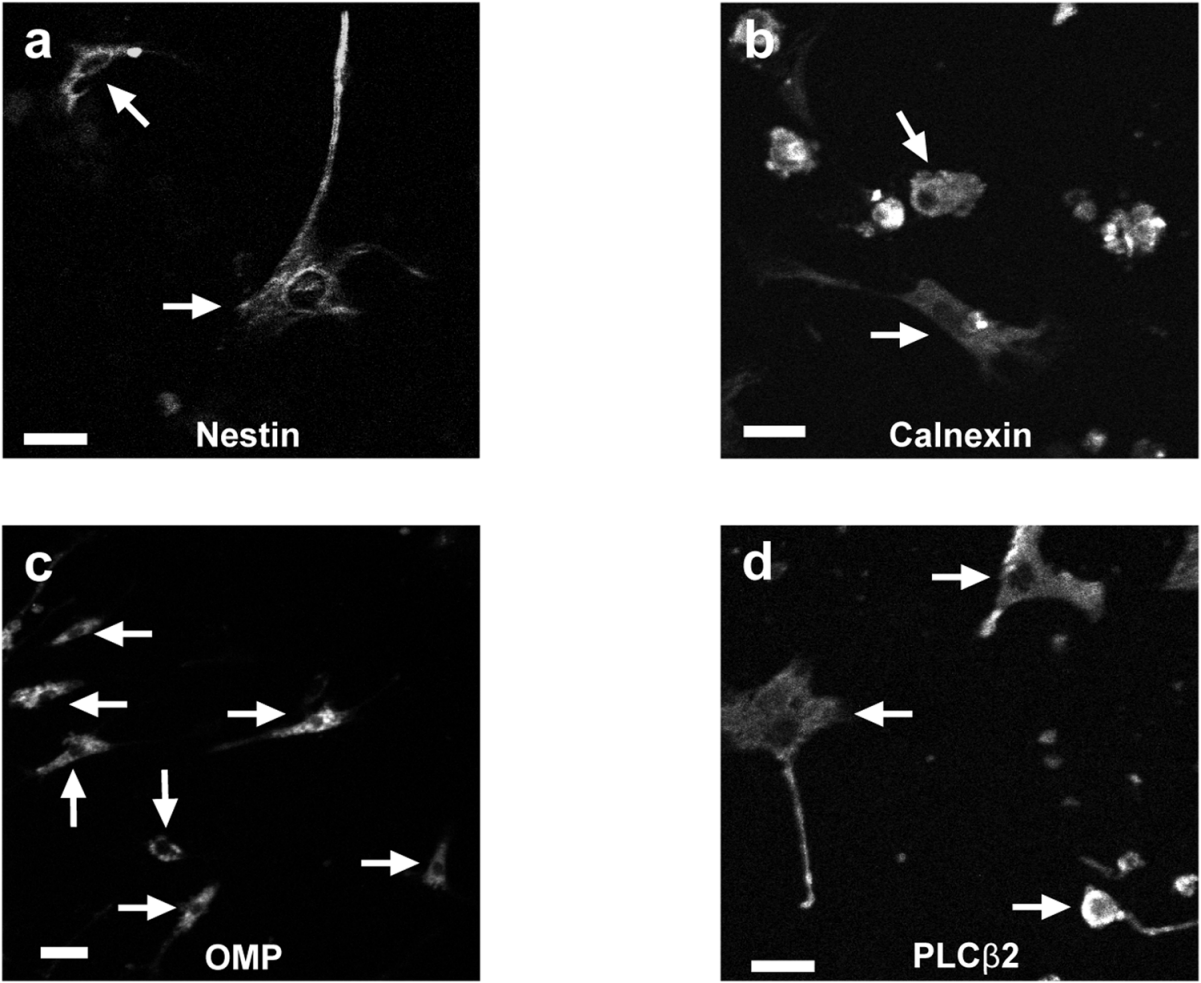
Immature OSN, mature OSN, sustentacular cells, and microvillous cells are present in 7–8 DIV adult OE primary epithelium cell cultures. (a–d). Representative grayscale pictures of cells present in adult primary olfactory cell cultures. Each panel corresponds to a separate immunocytochemistry experiment testing one antibody and showing cells immuno-positive for the tested antibody. (a) Use of antibody against neuronal precursor marker nestin, (b) use of antibody against sustentacular cell marker calnexin, (c) use of antibody against mature olfactory neuron marker OMP, and (d) use of antibody against microvillous cell marker PLCβ2. Scale bar = 20 µm. In each panel, each arrow indicates a single immunopositive cell; *n* = 3 coverslips for each tested antibody.

Primary cell cultures (7–8 DIV) were processed for immunoreactivity to rabbit anti-calnexin (1:500; Santa Cruz Biotechnology, Santa Cruz, CA), mouse anti-nestin (1:1,000; Millipore, Billerica, MA), goat anti-olfactory marker protein (OMP) (1:2,000; Wako Chemicals, Richmond, VA), or rabbit anti-phospholipase Cβ2 (PLCβ2) (1:100; Santa Cruz Biotechnology, Santa Cruz, CA). Cells were fixed in 4% paraformaldehyde for 20 min, washed with 0.1 M phosphate buffered saline (PBS), permeated with 0.05% tween 20 in 0.1 M PBS (PBST) for 10 min and blocked with 2% normal donkey serum (NDS) in PBST for 30 min. Cells were exposed to primary antibodies made in 2% NDS in PBST for 2 h at room temperature. Cy3-conjugated donkey anti-goat, fluorescein isothiocyanate (FITC)-conjugated donkey anti-rabbit, or tetramethylrhodamine (TRITC)-conjugated donkey anti-mouse (1:200; Jackson ImmunoResearch Labs, West Grove, PA) was applied for 30 min at room temperature. Sections were then washed and mounted in Vectashield mounting medium for fluorescence with 4′,6-diamidino-2-phenylindole (DAPI) (Vector Labs, Burlingame, CA) and visualized on an Olympus Fluoview 1,000 system. Cy3 dye was excited at 450 nm and low pass filtered at 560–620 nm. FITC dye was excited at 488 nm and low pass filtered at 505–525 nm. TRITC dye was excited at 543 nm and low pass filtered at 560–620 nm. DAPI was excited at 405 nm and fluorescence was detected at 430–470 nm. Antibody specificity was tested by omitting the primary antibodies or by using a peptide neutralization protocol in which the antibody (0.04 mg/mL) was combined with a 10-fold excess of the immunizing peptide (0.4 mg/mL). No immunoreactivity was observed in any of these controls. For each antibody, immunoreactive cells were tabulated based on size and optical density using the automated integrated morphometric analysis module from Metamorph software (Molecular Devices, Sunnyvale, CA) and expressed as a percent of total (DAPI-positive) cells; these cells are indicated by arrows in Figure 2. Positively-stained cells that could be dead or dying cells, or cell debris, were excluded from the tabulation (positively-stained cells not indicated by arrows in Figure 2).

### 2.8 Statistical analysis

We compared data sets with Prism Software version 9.1.0 (Graphpad Software, San Diego, CA). We used Student’s unpaired *t*-test to compare two data sets. To compare three or more data sets, if the standard deviation of any data set was not twice the value of the standard deviation of another data set, we used ordinary one-way ANOVA followed by Dunnett’s multiple comparison test to compare each data set to a control data set, or Tukey’s multiple comparison test, to make comparisons between all groups. If the standard deviation of any data set was at least twice the value of the standard deviation of any other data set, we used either Kruskal–Wallis test (if data sets failed the D’Agostino, Anderson–Darling, Shapiro–Wilk, and Kolmogorov–Smirnov normality tests) followed by Dunn’s multiple comparison test, or Brown–Forsythe and Welch ANOVA tests (if data sets did not fail the D’Agostino, Anderson–Darling, Shapiro–Wilk, and Kolmogorov–Smirnov normality tests) followed by Dunnett T3 multiple comparison test, for multiple comparisons correction [36,37]. A *p* value <0.05 was considered statistically significant.

## 3 Results

### 3.1 Cells in adult OE and OP6 cells express functional purinergic receptors

To conduct our study on ATP release in mouse adult OE, we decided to use adult OE tissue to characterize overall ATP release mechanisms regardless of the cell types involved, and OP6 cells to specifically characterize the contribution of OSN to ATP release. We used both undifferentiated OP6 cells that behave like immature OSN and differentiated OP6 cells that have the properties of mature OSN [33]. Part of our study requires us to determine whether an ATP-evoked ATP release mediated by stimulation of purinergic receptors, a mechanism that we characterized in neonatal mouse OE [4], is also present in adult OE. Therefore, we first verified that all preparations we were planning to use do express functional purinergic receptors, using calcium imaging. We chose adult OE primary cell cultures to determine whether adult OE as a whole does express functional purinergic receptors, since we had an established method of preparing and maintaining these cell cultures and using them for calcium imaging [38]. Application of 50 µM of non-specific purinergic receptor agonist ATP induced robust calcium responses in adult OE primary cell cultures and also in undifferentiated and differentiated OP6 cells (Figure 1; *n* = 3 coverslips and *n* = ≥7 cells for each preparation). ATP triggered an oscillatory calcium response in undifferentiated OP6 cells (Figures 1a–d) and a transient calcium response in differentiated OP6 cells (Figure 1b–e). In primary adult OE cell cultures (Figure 1c–f), ATP triggered either an oscillatory response (Figure 1f; black trace) or a transient response (Figure 1f; grey trace).

These data show that primary cell cultures from adult mouse OE tissue, undifferentiated OP6 cells, and differentiated OP6 cells all express functional purinergic receptors. While they might differ from cells present in whole intact adult OE, these preparations are suitable models to investigate what adult OE cell types express functional purinergic receptors and what cell types might contribute to purinergic receptor-mediated ATP release mechanisms in adult mouse OE. Furthermore, these results are in accordance with our previous report that both non-neuronal and neuronal cells cultured from the OE are responsive to purines [38]. However, the fact that our primary OE cell cultures responded to ATP application during calcium imaging does not show *per se* they contain all three cell types present in adult OE: OSN, sustentacular cells, and microvillous cells. We needed to confirm our cell cultures had all three cell types so that we could confidently conclude that OSN, sustentacular cells, and microvillous cells in adult OE express functional purinergic receptors, and that whole adult OE turbinates preparation can thus be used to study purine-evoked ATP release in subsequent experiments. To validate that our primary adult OE cell cultures had OSN, sustentacular cells, and microvillous cells, we used immunohistochemistry and tested four antibodies (one experiment for each tested antibody, and *n* = 3 coverslips for each tested antibody). We used antibodies against four markers: nestin, a neuronal precursor marker that would thus show the presence of the stem cells like basal cells; OMP, a marker of mature OSN; calnexin, a sustentacular cell marker; and PLCβ2, a microvillous cell marker. For each tested antibody, a fraction of the cells was immuno-positive (Table 1 and Figure 2).

**Table 1:** Percentage of immuno-positive cells in adult mouse OE primary cell cultures for each tested cell marker

**Percentage of immuno-positive cells in OE primary cell cultures (mean ± SEM) for each tested antibody**	
Nestin	8.16 ± 1.37
Calnexin	33.93 ± 9.42
OMP	49.43 ± 3.04
PLCβ2	40.94 ± 11.8

These positive immunocytochemistry results indicate that cells that responded to ATP application during calcium imaging of OE primary cell cultures and thus, expressed functional purinergic receptors, included all cell types present in the OE (basal cells, OSN, sustentacular cells, and microvillous cells). Thus, functional purinergic receptors are likely present in all cell types of adult OE tissue as well. Therefore, we validated that the whole adult OE turbinates are an adequate model to study mechanisms of purinergic receptor-mediated ATP release in adult mouse OE.

### 3.2 Purinergic receptor-mediated ATP release is present in adult OE but not in OP6 cells

**Figure 3:**
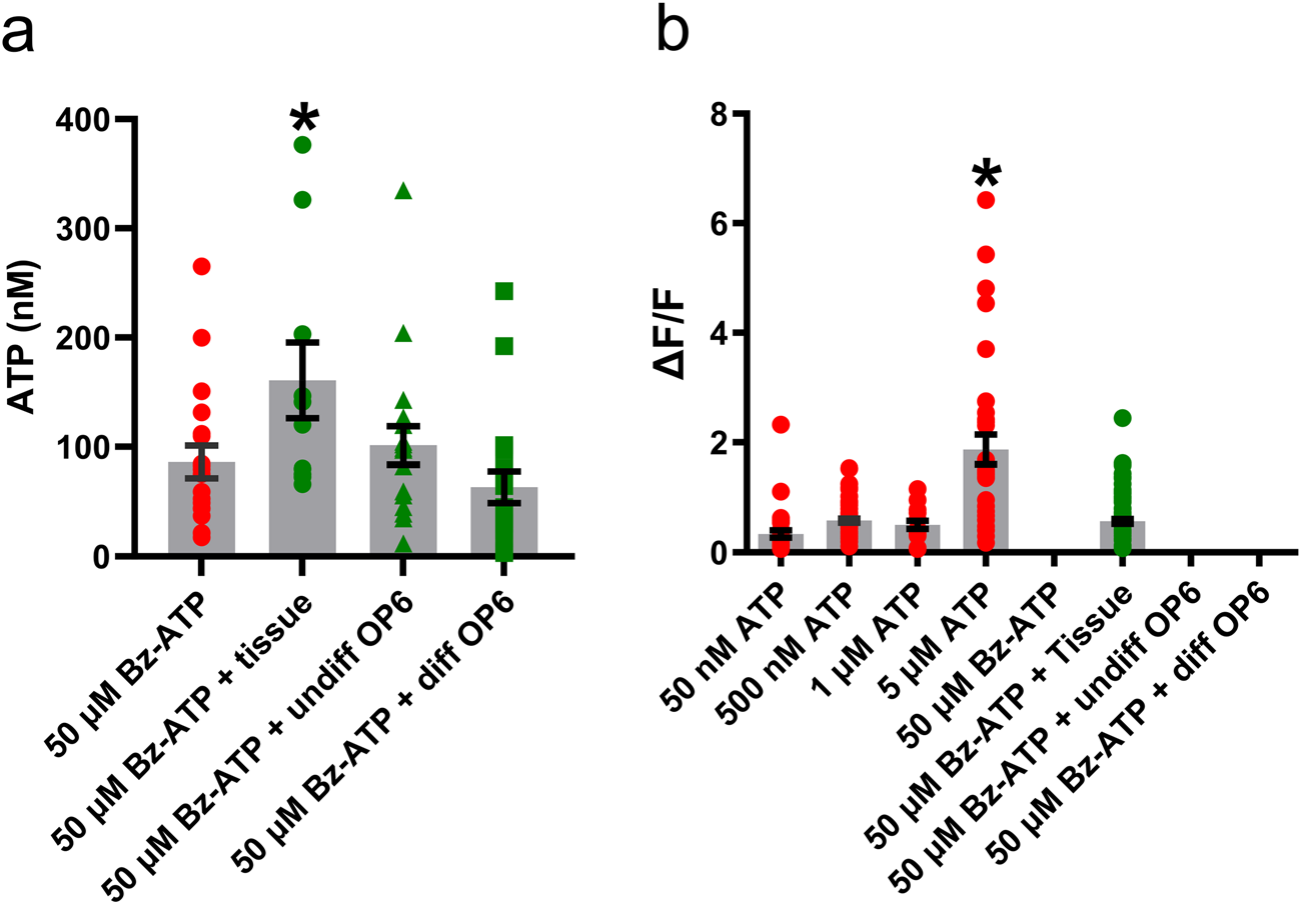
ATP-evoked release of ATP is present in adult OE but not in OP6 cells. (a) The luciferin and luciferase assay was used to quantify the amount of ATP (in nM; mean + SEM) released by undifferentiated OP6 cells (*n* = 18 assays; green triangles), differentiated OP6 cells (*n* = 19 assays; green squares), and adult OE turbinates (*n* = 10 assays; green circles) in presence of 50 µM purinergic receptor agonist Bz-ATP. Application of 50 µM Bz-ATP alone was used as a control (*n* = 19 assays; red circles). *: *p* < 0.05 vs 50 µM Bz-ATP (one-way ANOVA followed by Dunnett’s multiple comparison test). (b) Normalized fluorescence increase (mean + SEM) recorded during calcium imaging of transfected human embryonic kidney cells stably expressing P2X_2_ and used as ATP biosensors. Calcium responses were triggered in the biosensors by application of several ATP concentrations (red circles) and also by application of 50 µM Bz-ATP in presence of adult OE turbinates (*n* = 3 turbinates; green circles). Application of 50 µM Bz-ATP in absence of turbinates or in presence of undifferentiated or differentiated OP6 cells (*n* = 3 coverslips) did not trigger calcium responses in the biosensors. *: *p* < 0.05 vs any other mean (one-way ANOVA followed by Tukey’s multiple comparison test).

Characterization of ATP release mechanisms in adult mouse OE requires to investigate whether the evoked purinergic receptor-mediated ATP release we described in mouse neonatal OE [4] is also present in mouse adult OE cells. We quantified ATP release from whole adult OE turbinates using luciferin–luciferase assays, with 50 µM Bz-ATP as the purinergic receptor agonist. We also used both undifferentiated and differentiated OP6 cells to investigate whether immature and/or mature OSN could contribute to the evoked release of ATP. Bz-ATP was used to trigger an evoked ATP release since we previously showed that the amount of luminescence induced by application of 50 µM Bz-ATP alone is lower than the total luminescence produced by the evoked release of ATP triggered by this agonist [4]. Moreover, Bz-ATP is known to activate not only P2X_7_ receptors, but also several sub-types of P2Y receptors [39–42]. Statistical analysis of our data using one-way ANOVA followed by Dunnett’s multiple comparison showed the amount of bioluminescence measured when the agonist was applied in absence of cells or tissue was not significantly different compared to conditions where undifferentiated or differentiated OP6 were present (Figure 3a; *p* > 0.05). In contrast, the amount of bioluminescence detected when Bz-ATP was applied in presence of a set of turbinates was significantly higher compared to Bz-ATP alone (Figure 3a; *p* < 0.05), compared to Bz-ATP in presence of undifferentiated OP6 cells (Figure 3a; *p* < 0.05), and compared to Bz-ATP in presence of differentiated OP6 cells (Figure 3a; *p* < 0.05). For these experiments, the *n* value is ≥10 luciferin–luciferase assays for each group. To confirm that Bz-ATP can elicit an evoked release of ATP in adult OE turbinates, we ran experiments where we recorded calcium responses from transfected human embryonic kidney cells stably expressing P2X_2_ and used as ATP biosensors (*n* = 3 coverslips of biosensors and *n* ≥ 16 biosensors for each set of experiments). To show that the biosensors respond to the presence of ATP, calcium responses were recorded when they were perfused with varying ATP concentrations (50 nM, 500 nM, 1 µM, and 5 µM). The biosensors responded to all tested ATP concentrations (Figure 3b). Statistical analysis with one-way ANOVA followed by Tukey’s multiple comparison test showed the mean normalized fluorescence in presence of 5 µM ATP was significantly higher compared to 50 nM ATP (Figure 3b; *F*_(33,37)_ = 1.750; *p* < 0.05), compared to 500 nM ATP (Figure 3b; *F*_(33,60)_ = 1.630; *p* < 0.05), and compared to 1 µM ATP (Figure 3b; *F*_(33,16)_ = 2.178; *P* < 0.05). We then ran experiments where we imaged one coverslip of biosensors after application of 50 µM Bz-ATP alone, application of 50 µM Bz-ATP in presence of adult OE turbinates, application of 50 µM Bz-ATP in presence of one coverslip of undifferentiated OP6 cells, and application of 50 µM Bz-ATP in presence of one coverslip of differentiated OP6 cells. No calcium responses were triggered in the biosensors when Bz-ATP was applied alone, or in presence of undifferentiated or differentiated OP6 cells (Figure 3b). However, when Bz-ATP was applied in presence of turbinates, the biosensors responded with calcium increases, indicating that they detected ATP released by the turbinates following activation of the purinergic receptors located on the OE cells. Overall, the mean normalized fluorescence recorded in presence of the purinergic receptor-mediated ATP release was only significantly different when compared to the mean normalized fluorescence in presence of 5 µM of ATP (Figure 3b; *F*_(33,87)_ = 1.570; *p* < 0.05). Thus, the amount of ATP released by the adult OE turbinates when purinergic receptors were activated by Bz-ATP ranges from 50 nM to 1 µM.

These data confirm that a purinergic receptor-mediated evoked release of ATP does occur in adult OE. Undifferentiated and differentiated OP6 cells might differ from immature and mature OSN present in whole OE. Still, our data combining use of biosensors and OP6 cells still raise the possibility that immature and mature OSN do not contribute to the purinergic receptor-mediated ATP release present in adult mouse OE.

### 3.3 Characterization of the constitutive ATP release present in OP6 cells

**Figure 4:**
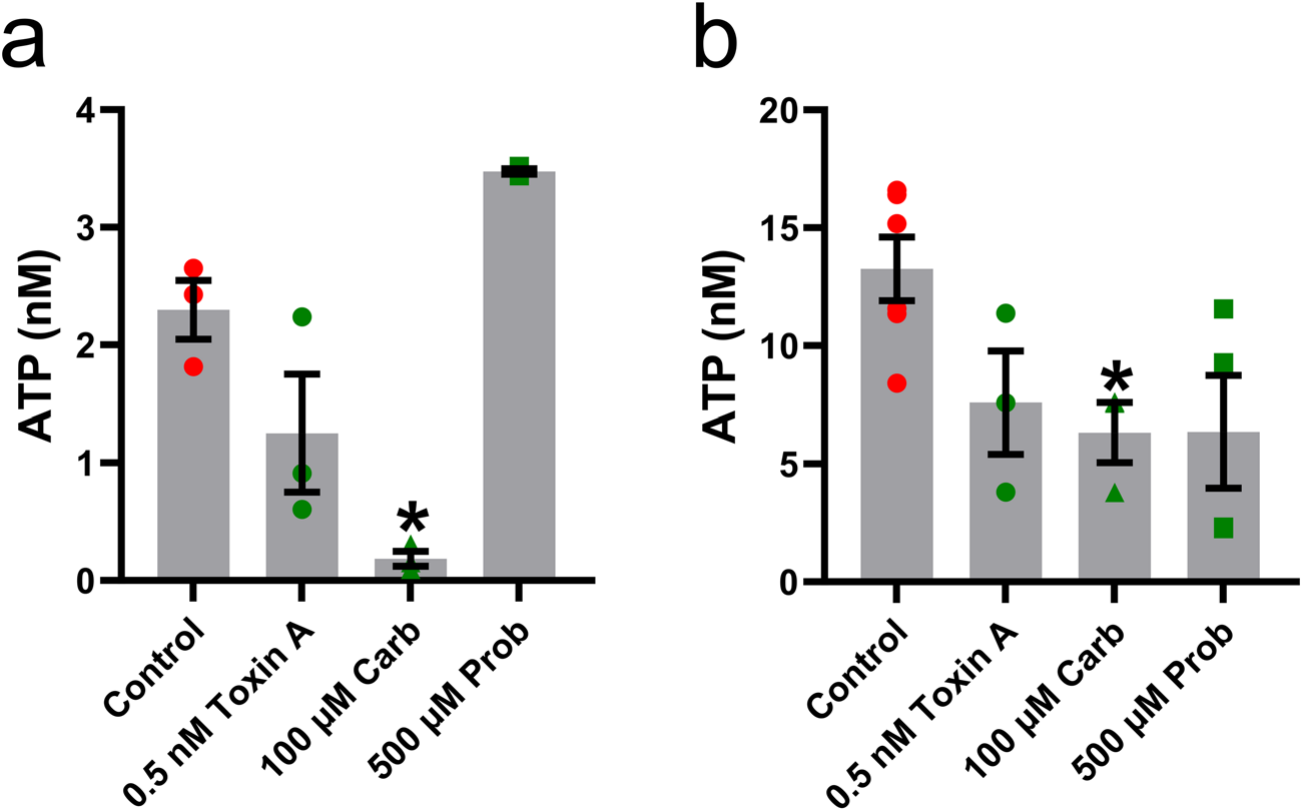
Constitutive ATP release characteristics in undifferentiated and differentiated OP6 cells. The luciferin and luciferase assay was used to quantify the amount of ATP (in nM; mean + SEM) released by undifferentiated OP6 cells (*n* = 3 assays for each group; a) and differentiated OP6 cells (*n* = 3–6 assays for each group; b) in absence of any inhibitors (control; red circles), in presence of 0.5 nM vesicular fusion inhibitor *Clostridium difficile* toxin A (green circles), 100 µM carbenoxolone, an inhibitor of connexin hemichannels and pannexin channels (green triangles), or 500 µM ABC transporter inhibitor probenecid (green squares). *: *p* < 0.05 vs control (Brown–Forsythe and Welch ANOVA tests followed by Dunnett T3 multiple comparison test).

Lack of purinergic receptor-mediated ATP release in OP6 cells does not mean absence of ATP release from these cells. Indeed, we showed the presence of a constitutive ATP release occurring in neonatal OE and driven by constitutive calcium-independent exocytosis, connexin and/or pannexin1 hemichannels, and ATP binding cassette (ABC) transporters [4]. To investigate whether similar mechanisms are present in OP6 cells, we used luciferin–luciferase assays. During these assays, coverslips of either undifferentiated or differentiated OP6 cells were placed in control Ringer’s solution, Ringer’s solution containing 0.5 nM vesicular fusion inhibitor *Clostridium difficile* toxin A, Ringer’s solution containing 100 µM of non-specific connexin and pannexin inhibitor carbenoxolone, or Ringer’s solution containing 500 µM probenecid, an inhibitor of pannexin1 hemichannels and of ABC transporters (*n* ≥ 3 coverslips for each group of experiments). Statistical analysis using Brown–Forsythe and Welch ANOVA tests followed by Dunnett T3 multiple comparison showed carbenoxolone was the only treatment to significantly impact the amount of bioluminescence, inducing a decrease compared to control Ringer’s conditions (Figure 4a; *p* < 0.05). Similar results were observed when we used differentiated OP6 cells (Figure 4b; *p* < 0.05). Overall, these data obtained from OP6 cells raises the possibility that constitutive ATP release does occur in immature and mature OSN in adult OE, via ATP efflux through connexin hemichannels. Additionally, the amount of ATP released in control conditions from undifferentiated OP6 cells was significantly lower than the amount of ATP released in control conditions from differentiated cells (2.3 ± 0.25 vs 13.26 ± 1.35; *p* < 0.05, unpaired two-tailed Student’s *t*-test) indicating that OSN release more ATP as they mature. The difference in the responsiveness to carbenoxolone and probenecid treatments between panels a and b could be explained by the difference in OP6 cell maturation.

### 3.4 Characterization of the constitutive and evoked ATP release present in adult mouse OE turbinates

**Figure 5:**
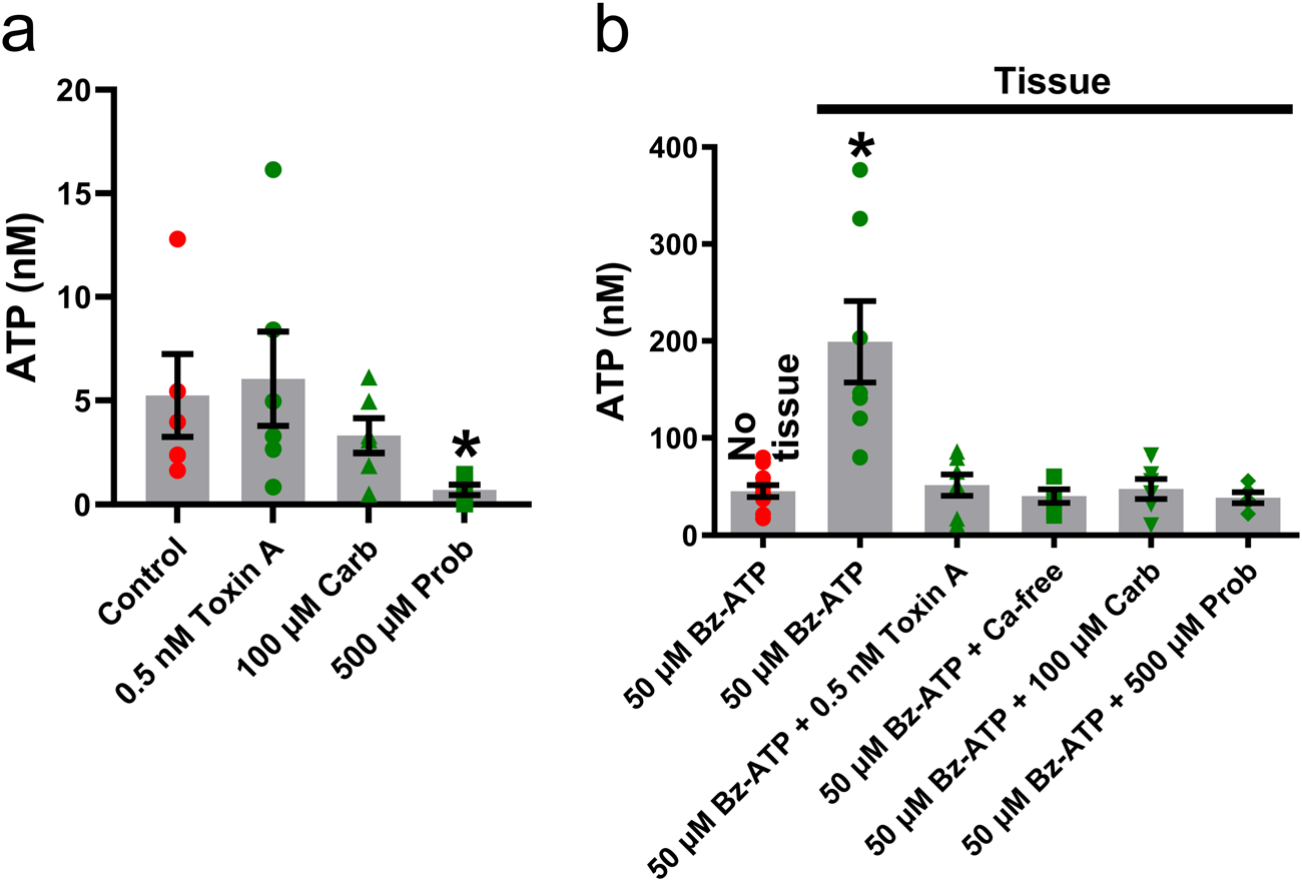
Constitutive and ATP-evoked ATP release characteristics in adult whole OE turbinates. (a) The luciferin and luciferase assay was used to quantify the amount of ATP (in nM; mean + SEM) released from adult turbinates by the constitutive release of ATP in absence of any inhibitors (control red circles), in presence of 0.5 nM vesicular fusion inhibitor *Clostridium difficile* toxin A (green circles), 100 µM carbenoxolone, an inhibitor of connexin hemichannels and pannexin channels (green triangles), or 500 µM ABC transporter inhibitor probenecid (green squares). *n* = 5–6 assays for each group. *: *p* < 0.05 vs control (Kruskal–Wallis test). (b) The luciferin and luciferase assay was used to quantify the amount of ATP (in nM; mean + SEM) released from adult turbinates by the evoked release of ATP triggered by application of 50 µM Bz-ATP (green symbols). Essays were done in Ringer’s solution alone (50 µM Bz-ATP; green circles), in presence of 0.5 nM vesicular fusion inhibitor *Clostridium difficile* toxin A (green triangles), calcium-free Ringer’s solution (green squares), 100 µM carbenoxolone, an inhibitor of non-specific pannexin and activated P2X_7_ receptor inhibitor (green inverted triangles), or 500 µM selective pannexin 1 channel and ABC transporter inhibitor probenecid (green diamonds). Application of 50 µM Bz-ATP in Ringer’s solution in absence of adult turbinates was used as a control (red circles). *n* = 5–11 assays for each group. *: *p* < 0.05 vs control (Brown–Forsythe and Welch ANOVA tests followed by Dunnett T3 multiple comparison test).

Results obtained from luciferin–luciferase assays and use of biosensors showed that Bz-ATP elicited a purinergic receptor-mediated evoked release of ATP in adult mouse OE turbinates. However, constitutive release of ATP might also be present in adult mouse OE, similar to what occurs in neonatal mouse OE [4], and could be detected via luciferin–luciferase assays. We used the same experimental conditions as the one used for the OP6 cells study (Figure 5a), with one set of turbinates per assay, and *n* ≥ 5 assays for each group. Statistical analysis using Kruskal–Wallis test showed that 500 µM probenecid was the only treatment to significantly impact the mean bioluminescence, significantly decreasing it compared to control Ringer’s condition (Figure 5a; *p* < 0.05). Therefore, the overall constitutive release of ATP occurring in adult mouse OE through all involved cell types is mediated by ABC transporters. We then aimed to characterize the precise mechanisms of the evoked release of ATP that we revealed in adult mouse OE turbinates. The ATP-evoked ATP release we characterized in mouse neonatal OE occurs via several mechanisms: (a) calcium influx through activated P2X_7_ receptors and followed by calcium-dependent exocytosis (b) through activated P2X_7_ receptor–pannexin1 complexes, and (c) via calcium-dependent exocytosis following P2Y purinergic receptor stimulation [4]. We used luciferin–luciferase assays to test whether similar mechanisms underlie the evoked release of ATP triggered by Bz-ATP in adult mouse OE. We hypothesized that the evoked release of ATP would be inhibited by use of vesicular fusion inhibitor *Clostridium difficile* toxin A, use of calcium-free Ringer’s solution, use of Ringer’s solution containing non-specific connexin and pannexin inhibitor carbenoxolone, or Ringer’s solution containing probenecid, a specific inhibitor of pannexin1 hemichannels. For each assay, a single set of turbinates was put into Ringer’s solution and stimulated with 50 µM Bz-ATP in presence or absence of one of the inhibitors, and application of 50 µM Bz-ATP in absence of turbinates was used as control assays (*n* ≥ 5 assays for each group). Statistical analysis using Brown–Forsythe and Welch ANOVA tests followed by Dunnett T3 multiple comparison test showed stimulation of turbinates with Bz-ATP in absence of any inhibitor significantly increased the amount of bioluminescence compared to control (Figure 5b; *p* < 0.05). Use of 0.5 nM *Clostridium difficile* toxin A significantly decreased the amount of bioluminescence compared to application of Bz-ATP in presence of tissue (Figure 5b; *p* < 0.05). Use of calcium-free Ringer’s had a similar effect (Figure 5b; *p* < 0.05), as well as use of 100 µM carbenoxolone (Figure 5b; *p* < 0.05), or 500 µM probenecid (Figure 5b; *p* < 0.05).

Our results show that the purinergic receptor-mediated evoked release of ATP in adult mouse OE occurs via mechanisms similar to part of the evoked release of ATP occurring in neonatal mouse OE. Namely, calcium influx through activated P2X_7_ receptors followed by calcium-dependent exocytosis of ATP, and ATP efflux through activated P2X_7_ receptor/pannexin1 complexes [4].

## 4 Discussion

Constitutive release of ATP occurs under physiological conditions in many organ systems [43]. Exogenous ATP, a simulation of injury, evokes the release of ATP in many cell types [20–23]. We previously revealed the presence of both constitutive and ATP-evoked ATP release in neonatal mouse OE [4] and extensively demonstrated how both types of ATP release play a role in constant mouse OE homeostasis and post-injury neuroregeneration, including ATP-induced release of other neurotrophic factors [2,4,11,12,14–18]. The evoked release of ATP in neonatal mouse OE includes pathways downstream of P2X_7_ and P2Y purinergic receptors stimulation [4]. We hypothesized that the adult mouse OE has mechanisms of ATP release similar to the ones present in neonatal mouse OE (e.g., constitutive and activated purinergic receptor-evoked ATP release) and tested this hypothesis using luciferin–luciferase assays and ATP biosensors. We did reveal the presence of both constitutive and activated purinergic receptor-mediated ATP release in adult mouse OE whole turbinate preparations. This is in accordance with our calcium imaging data showing the presence of functional purinergic receptors in adult OE primary cell cultures. This preparation is adequate to study calcium responses in adult mouse OE, since immunostaining showed all OE cell types in our cell cultures. We also investigated the contribution of a specific OE cell type, the OSN, to both releases of ATP, by using the OP6 cell line. Undifferentiated OP6 cells are similar to immature OSN, and differentiated OP6 cells are similar to mature OSN. Both immature and mature OSN displayed constitutive ATP release, but lacked a purinergic receptor-mediated one during our experiments; this absence is not due to absence of functional purinergic receptors in OP6 cells. Indeed, calcium imaging showed that both undifferentiated and differentiated OP6 cells do respond to ATP application, like our adult primary OE cell cultures. Therefore, either OSN only constitutively release ATP, or their putative evoked release of ATP does not involve receptors that can be activated by Bz-ATP.

When we investigated the precise mechanisms underlying both types of ATP release, we showed that the constitutive release of ATP in immature and mature OSN occurs via connexin and/or pannexin hemichannels. It is in accordance with the presence of both connexin and pannexin hemichannels in the central nervous system [44]. Furthermore, hemichannels are present in the OE [45] and it has been shown that both mature and immature OSN express the connexin 43 subunit [46]. Surprisingly, the constitutive release of ATP in our adult OE turbinate preparations was not inhibited by carbenoxolone, a non-specific connexin and pannexin inhibitor. An explanation is that the ATP released by OSN in adult mouse OE represents a minimal amount of the total ATP being released by the whole OE. Thus, the carbenoxolone treatment would not inhibit enough ATP release to have a significant effect. Our data support this explanation, since probenecid, which inhibits ABC transporters, significantly inhibited the constitutive release of ATP in the OE turbinate preparations. It shows that the main mechanism underlying the constitutive release of ATP in adult OE is ATP efflux through ABC transporters. It is coherent with the fact that we showed the same mechanism in neonatal mouse OE [4]. It was surprising that vesicular fusion inhibitor *Clostridium difficile* toxin A did not inhibit the constitutive ATP release in adult mouse OE. Indeed, we showed this inhibitor blocked part of the constitutive ATP release in neonatal mouse OE happening via constitutive calcium-independent exocytosis [4], a type of exocytosis that has been documented in other models as well [47–49]. This discrepancy can be explained by changes in mechanisms of ATP release when the OE matures from neonatal to adult. Indeed, it has been shown that exocytosis might be affected by age-related changes [50,51]. Moreover, our data from OP6 cells showed that OSN release more ATP as they mature, further supporting this explanation. Our results showed the evoked release of ATP occurring after purinergic receptor stimulation in adult OE shares mechanisms with the one present in neonatal mouse OE [4]: influx of calcium ions through activated P2X_7_ receptors followed by calcium-dependent exocytosis, and efflux of ATP through activated P2X_7_–pannexin1 complexes. Indeed, it was inhibited by use of (a) vesicular fusion inhibitor *Clostridium difficile* toxin A and calcium-free Ringer’s, conditions that inhibit calcium influx and calcium-dependent exocytosis and (b) use of non-specific connexin and pannexin inhibitor carbenoxolone and use of selective pannexin1 inhibitor probenecid, conditions that inhibit activated P2X_7_–pannexin1 complexes. While Bz-ATP can also activate some P2Y receptors besides P2X7 receptors, the fact that use of calcium-free Ringer’s totally abolished the evoked release of ATP suggests that this release occurs in adult OE solely via P2X_7_ receptors and does not involve P2Y receptors. Bz-ATP would still have induced a significant release of ATP in calcium-free Ringer’s if part of the evoked release of ATP in adult OE was mediated by calcium-dependent exocytosis of ATP following P2Y purinergic receptor stimulation. This is surprising since we showed P2Y receptor stimulation is involved in the evoked release of ATP occurring in neonatal mouse OE [4]. An explanation is that the mechanisms underlying evoked ATP release change during maturation of the OE. Another explanation is that the P2Y receptors involved in the evoked ATP release in adult OE are not activated by Bz-ATP. Further studies using P2Y receptor agonists besides Bz-ATP are required to explain this discrepancy between neonatal and adult evoked ATP release. Moreover, calcium influx through other types of activated P2X receptor subtypes besides P2X_7_ might also contribute to the evoked ATP release in adult mouse OE, since multiple P2X and P2Y receptor subtypes have been reported in mouse OE [2–4].

**Figure 6:**
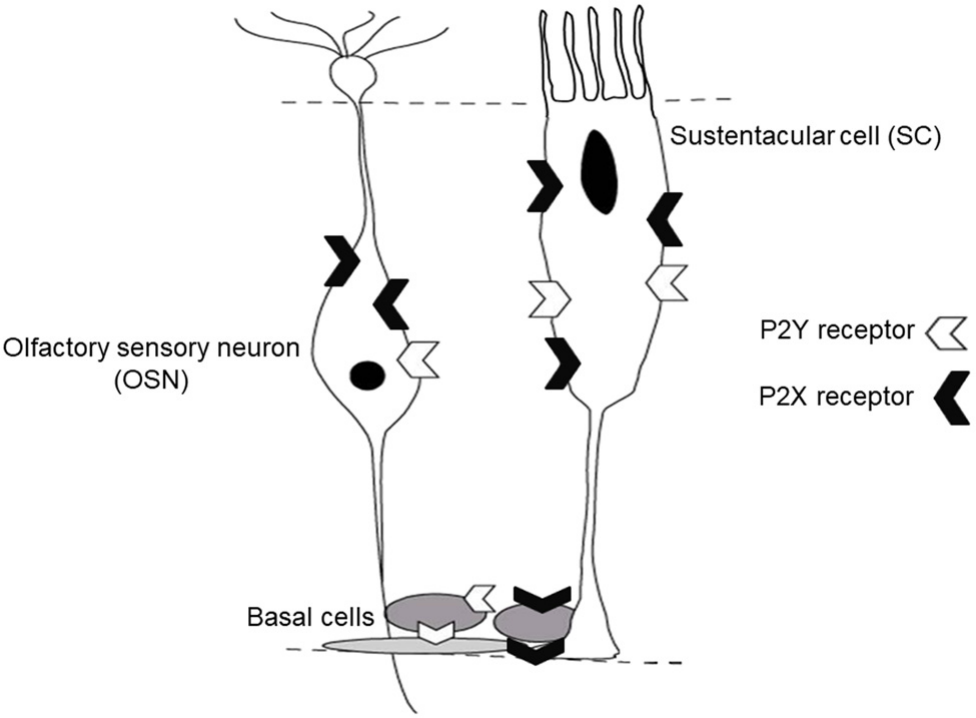
Schematic of the organization of the OE. The cell bodies of the glial-like sustentacular cells make the apical layer of the OE; the cell bodies of the OSN make the middle layer of the OE; and the stem cells-like horizontal and basal cells make the basal layer of the OE. All cell types express P2X and P2Y purinergic receptors.

Now, a burning question is: what OE cell types are involved in constitutive and/or evoked ATP release in adult mouse OE? As shown in Figure 6, glial-like sustentacular cells are located in the apical layer of the OE; the OSN are located in the OE middle layer; and the basal layer of the OE houses the horizontal and the basal cells of the OE. The OE middle layer also houses microvillous cells. While their exact function is still unknown, we previously showed that, in neonatal OE, these cells express both IP3R3 receptors and neurotrophic factor NPY and that they are involved in tissue homeostasis and regeneration [18].

**Figure 7:**
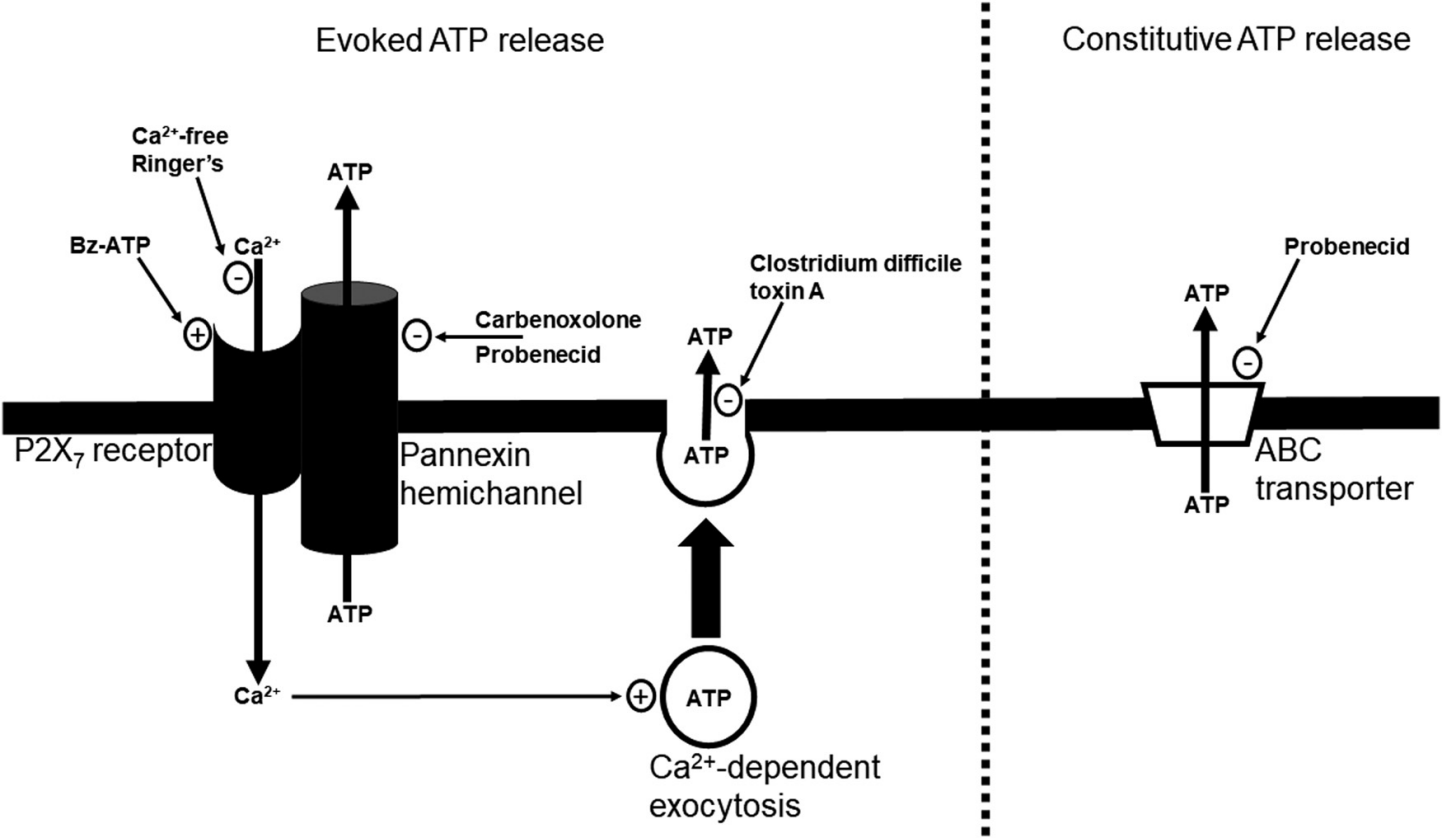
Schematic of mechanisms underlying the evoked ATP release and the constitutive ATP release. Extracellular ATP, leaking from damaged cells or released constitutively, activates P2X_7_ purinergic receptors, leading to Ca^2+^ influx into the cell via activated P2X_7_ receptors and subsequent Ca^2+^-dependent exocytosis of ATP. Activated P2X_7_ receptors also form a complex with pannexin hemichannels through which ATP can efflux. The constitutive ATP release is mediated by ABC transporters. Inhibitors (−) and stimulators (+) are indicated in the figure.

Could sustentacular cells, microvillous cells, and OSN all be involved in the ATP release mechanisms we describe in the present study and summarize in Figure 7?

Our data rule out the presence of ATP efflux via ABC transporters in OSN but also shows that the main amount of constitutively released ATP from whole OE turbinates comes from ABC transporters action. Thus, the ABC transporter-mediated mechanism of constitutive ATP release has to occur in sustentacular and/or microvillous cells. The lack of ATP release mediated by Bz-ATP in OP6 cells means that sustentacular cells and/or microvillous are most likely responsible of the evoked release of ATP present in adult mouse OE. The fact that RNAseq databases showed P2rx7 is a non-OSN gene [52] supports this theory. It would make sense since astrocytes are known to release ATP via exocytosis [53,54] and sustentacular cells are the glia-like cells of the OE. Moreover, it has been well documented that vesicles, dense bodies, and secretory granules are present in the cytosol of sustentacular cells [55–59]. Sustentacular cells also display calcium store-mediated signaling [60]. The microvillous cells might also contribute to the evoked release of ATP via calcium-dependent exocytosis. They express IP3R3 receptors, a component of calcium-dependent exocytosis, and release neurotrophic factor NPY upon stimulation with ATP and UTP, two purinergic receptor agonists that lead to intracellular calcium increase, a requirement for calcium-dependent exocytosis [15,18]. Use of transgenic mice where specific reporters are used to label a specific OE cell type, like the IP3R3-tauGFP mouse we used in a previous study [18], are tools that could allow to precisely identify what OE cell type is involved in the constitutive and/or the evoked ATP release.

Our results suggest that sustentacular and/or microvillous cells are mostly responsible for both the constitutive and evoked release of ATP, OSN only minimally contributing to the constitutive release of ATP. Our results show that P2X_7_ receptors are definitely involved in the evoked release of ATP present in adult OE. Other purinergic receptors, including P2Y receptors might be involved as well. We are well aware of the limitations of our study. Our OP6 cells are a model to study OSN but are not OSN *per se*. Likewise, our primary OE cell cultures are not undissociated adult OE tissue *per se*. Also, probenecid inhibits both ABC transporters and pannexin hemichannels. If, in our characterization of the constitutive ATP release, probenecid and carbenoxolone would have inhibited the same target, the data sets for carbenoxolone and probenecid would have been similar in both panels of Figure 4 and in panel a of Figure 5. However, our luciferin–luciferase assays showed the effects of carbenoxolone and probenecid on the constitutive ATP release differ, enabling us to propose connexin hemichannels activity underlie the constitutive ATP release in OP6 cells, while ABC transporters activity is mainly responsible for the constitutive ATP release in whole adult OE. Also, the fact both probenecid and carbenoxolone target and inhibit the activated P2X_7_ receptor/pannexin hemichannel complex during the evoked release of ATP was not detrimental to our study, since it enabled us to confirm this complex is indeed involved in the evoked ATP release in adult mouse OE. We did calcium imaging of primary OE cell cultures because we do not have expertise in calcium imaging of whole intact adult OE turbinates, a method that has been successfully used by other laboratories [61]. Despite these limitations, our study revealed the presence of constitutive and purinergic receptor-mediated ATP release mechanisms in adult mouse OE. We thus paved the way for future projects aiming to characterize even more the mechanisms of ATP release in adult mouse OE, which could involve intact adult OE turbinates calcium imaging. Olfactory ensheathing glia (OEG) is another cell type that might also contribute to ATP release and the cell turnover and post-injury neuroregeneration mechanisms present in adult mouse OE. Indeed, these cells are found in association with the olfactory nerve [62]. Moreover, these cells contribute to regeneration of the primary olfactory nervous system and OEG transplantation promotes regeneration in the spinal cord, the visual system, and the central nervous system [63–66]. Thus, the putative contribution of OEG to the constitutive and/or evoked release of ATP present in adult mouse OE should be investigated. These future studies are required to determine what adult mouse OE cell type is involved in each kind of release, what purinergic receptor subtypes activate the evoked release of ATP, and how the two ATP releases are involved in OE homeostasis and post-injury neuroregeneration.
